# *Transactions of the American Surgical Association*. Vol. II.

**Published:** 1885-06

**Authors:** 


*Transactions of the American Surgical Association.* Vol. II., 1885. Pp. 531. Philadelphia: Blakiston & Co.

Judging from the contents of the handsome volume now before us, we augur a brilliant future for the young American Surgical Association. There are some six and twenty original papers collected here, with short reports of the discussions which they gave rise to. Some of these papers are of quite unusual excellence, and there is not one which is unworthy of the position and the permanence which this publication must give. The first paper, on "Wounds of the Intestines," by the late Professor S. D. Gross, was read by Dr. Richardson, while the "Nestor of American Surgery" was on his death-bed, and is characterised by the usual thoroughness and insight of this master in his art. Papers on Anæsthetics; on surgical interference in Cerebral Abscess, in Epilepsy, and in Insanity; various clinical investigations on different subjects; and some valuable original studies, follow and make up the volume. Among the original memoirs, we must mention one by Dr. N. Senn, of Milwaukee, on "Cicatrization in Blood-vessels after Ligature," as being quite the most important recent contribution to our knowledge of this all-important subject. Altogether, it would be difficult to select from among the numerous "Transactions" of our various societies one which could surpass or even equal in practical value this one of the American Surgical Association.

